# Association between Serum IGF-I levels and Postoperative Delirium in Elderly Subjects Undergoing Elective Knee Arthroplasty

**DOI:** 10.1038/srep20736

**Published:** 2016-02-05

**Authors:** Timothy E. Yen, John C. Allen, Sarah K. Rivelli, Stephanie C. Patterson, Meredith R. Metcalf, Benjamin J. Flink, Aibek E. Mirrakhimov, Sandhya A. Lagoo, Thomas P. Vail, Christopher C. Young, Richard E. Moon, Paula T. Trzepacz, Madan M. Kwatra

**Affiliations:** 1 Duke-NUS Graduate Medical School, Singapore; 2 Duke University School of Medicine, Department of Psychiatry, Durham, NC 27710; 3 Duke University School of Medicine, Durham, NC 27710; 4 University of Missouri - Kansas City, Department of Surgery, Kansas City, MO 64108; 5 Emory University School of Medicine, Department of Surgery, Atlanta, GA 30307; 6 Saint Joseph Hospital, Department of Internal Medicine, Chicago, IL 60657; 7 Duke University School of Medicine, Department of Surgery, Durham, NC 27710; 8 University of California, San Francisco, San Francisco, CA 94143; 9 Duke University School of Medicine, Department of Anesthesiology, Durham, NC 27710; 10 Indiana University School of Medicine, Indianapolis, IN 46202; 11 Tufts University Medical School, Boston, MA 02111.

## Abstract

Evidence is mixed for an association between serum insulin-like growth factor-I (IGF-I) levels and postoperative delirium (POD). The current study assessed preoperative serum IGF-I levels as a predictor of incident delirium in non-demented elderly elective knee arthroplasty patients. Preoperative serum levels of total IGF-I were measured using a commercially available Human IGF-I ELISA kit. POD incidence and severity were determined using DSM-IV criteria and the Delirium Rating Scale-Revised-98 (DRS-R98), respectively. Median IGF-I levels in delirious (62.6 ng/ml) and non-delirious groups (65.9 ng/ml) were not significantly different (p = 0.141). The ratio (95% CI) of geometric means, D/ND, was 0.86 (0.70, 1.06). The Hodges-Lehmann median difference estimate was 7.23 ng/mL with 95% confidence interval (−2.32, 19.9). In multivariate logistic regression analysis IGF-I level was not a significant predictor of incident POD after correcting for medical comorbidities. IGF-I levels did not correlate with DRS-R98 scores for delirium severity. In conclusion, we report no evidence of association between serum IGF-I levels and incidence of POD, although the sample size was inadequate for a conclusive study. Further efforts to investigate IGF-I as a delirium risk factor in elderly should address comorbidities and confounders that influence IGF-I levels.

Delirium is characterized by fluctuating disturbances in attention, memory, orientation, perception, psychomotor behavior, and sleep. Postoperative delirium (POD) is a common adverse outcome in patients after major surgery with incidence of up to 65%^1^. Older patients who develop POD are at greater risk for associated short-term and long-term negative sequelae. Acutely, POD has been linked to higher rates of inpatient mortality and morbidity, in addition to longer hospital stays and higher hospitalization costs^2^,^3^,^4^. The long-term effects of POD in the elderly include neurological impairment^5^,^6^,^7^, functional decline^8^, and increased mortality^9^.

The etiology and pathogenesis of delirium is complex and not well understood. From a clinical standpoint, known delirium risk factors include age, previous cognitive impairment, dementia, sleep deprivation, opioid pain treatment, exposure to benzodiazepines, and obstructive sleep apnea^10^,^11^. On a cellular level, inflammation, oxidative stress, apoptosis, and alterations in neurohormonal signaling underlie the clinical manifestations of this disorder^12^. There are ongoing efforts to better understand the molecular basis of delirium in order to gain insight into the direct pathophysiology of the disease and to identify blood predictors to aid in clinical risk determination and diagnosis.

Insulin-like growth factor-I (IGF-I) is being investigated as a possible delirium risk predictor. IGF-I is believed to be neuroprotective, and low IGF-I levels have been associated with aging and Alzheimer’s dementia, both of which are major risk factors for delirium^12^,^13^,^14^,^15^. Thus, it is possible that low levels of IGF-I could predispose to the development of POD. However, the evidence remains uncertain. Six research groups have investigated the association between circulating IGF-I levels and the onset of delirium in response to surgery or acute medical illness^16^,^17^,^18^,^19^,^20^,^21^,^22^. Three authors have found a significant association between low levels of IGF-I and delirium^18^,^19^,^21^,^22^, while three others did not^16^,^17^,^20^. Potential reasons for this discrepancy include small patient cohorts and different study designs, IGF-I assay methodology, patient exclusion criteria, patient health status (acutely ill vs healthy), and clinical status (postoperative vs medically ill).

In order to better understand the relationship between preoperative serum IGF-I and POD without the confound of dementia, we prospectively studied 106 healthy, non-demented elderly subjects undergoing elective knee replacement surgery about whom we previously reported a POD incidence of 27%^23^. We hypothesized that subjects with lower levels of plasma IGF-I would be more likely to develop delirium. Indeed, we previously presented a preliminary analysis of 85 subjects from this cohort that found a statistically significant association between lower preoperative plasma IGF-I levels and increased incidence and severity of POD^24^. We now report our final analysis (n = 98).

## Results

### Baseline Comparisons

Table 1 shows clinical characteristics at baseline comparing delirious (D) (n = 22) and non-delirious (ND) (n = 76) groups. There were no significant differences between D and ND groups for demographic characteristics, preoperative hemoglobin, oxygen saturation, or medical comorbidities except for obstructive sleep apnea, which was more common in the D group (ND, 9.2%; D, 31.8%; p = 0.014).

**Figure 1:**
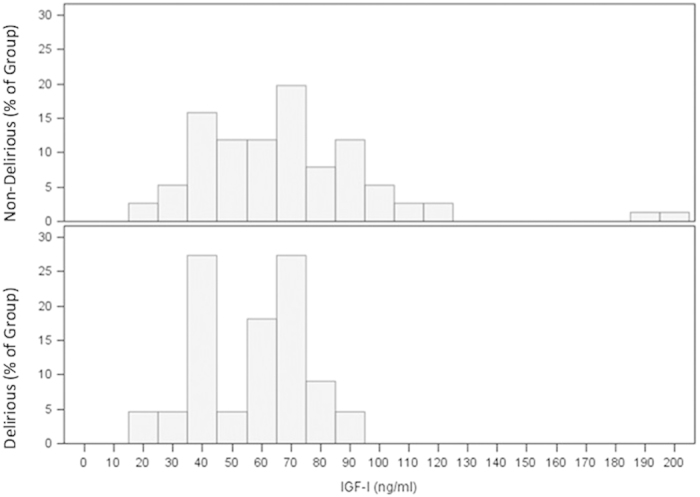
Distributions of preoperative IGF-I serum levels by percentage for delirious and non-delirious groups. The distributions of IGF-I concentrations for the Delirious and Non-delirious groups exhibited considerable overlap, and there was no difference in median IGF-I levels between groups.

The distributions of IGF-I levels for the D and ND groups exhibited considerable overlap (Fig. 1). Mean (SD) preoperative serum IGF-I levels (ng/mL) were 57.0 (18.6) in the D group and 68.4 (31.6) in the ND group. Study groups were compared using a 2-sample t-test after taking logarithms, resulting in a non-significant difference (p = 0.146). Geometric means (GSD) were ND: 62.3 (1.10) and D: 53.6 (1.07). GSDs were sufficiently similar to warrant a pooled estimate in calculating a confidence interval on the GM ratio. The GM ratio and 95% CI for D/ND was 0.86 and (0.70, 1.06) indicating that mean preoperative IGF-I level in delirium subjects was 14% lower than in non-delirium subjects and could be as much as 30% lower or 6% higher. Study group medians were ND: 65.9 and D: 62.6 ng/mL which were not significantly different by the Wilcoxon rank-sum test (p = 0.141). The Hodges-Lehmann median difference estimate was 7.23 ng/mL with 95% confidence interval (−2.32, 19.9).

### POD Prediction Analyses

Nineteen clinical baseline variables (Table 1) were assessed as potential predictors of POD using univariate logistic regression. Variables significant at p ≤ 0.15 were obstructive sleep apnea (p = 0.012), IGF-I concentration (p = 0.108), and diabetes (p = 0.147).

Multivariate regression analysis also resulted in IGF-I as a non-significant risk factor for POD (OR = 0.985, 95% CI: 0.964, 1.007; p = 0.183) after adjustment for obstructive sleep apnea (p = 0.024) and diabetes (p = 0.273) (Table 2).

Presence of obstructive sleep apnea increased the odds of developing POD by a factor of 4.60 (95% CI 1.40–15.1; p = 0.012) in the univariate logistic regression analysis and by 4.06 (95% CI; 1.21–13.7; p = 0.024) after adjustment for IGF-I and diabetes. Univariate area under the curve (AUC) (95% CI) for obstructive sleep apnea was 0.61 (0.51, 0.72). As previously reported, obstructive sleep apnea remained the only significant, independent predictor of POD in the multivariate analyses^23^.

### Delirium Severity

As expected, mean DRS-R98 scores were significantly different for delirious and non-delirious groups for Total (D: 16.1 ± 5.73, ND: 3.70 ± 3.62; p < 0.0001) and Severity scale scores (D: 10.7 ± 4.98, ND: 2.07 ± 2.05; p < 0.0001). No preoperative clinical variable studied in the univariate or multivariate regression analysis was significantly associated with delirium severity.

## Discussion

We report our final study results on the evaluation of serum IGF-I as a potential predictor of risk of post-operative incident delirium in 98 healthy, non-demented elderly patients who underwent elective knee surgery. Our study team previously presented interim results from this cohort that found a significant association between lower preoperative plasma IGF-I levels and both increased incidence (p = 0.012) and severity (p = 0.018) of POD^24^. However, we did not find a significant relationship in this final report. One notable difference between our previous, unpublished abstract and this current report is that we measured serum IGF-I levels instead of plasma IGF-I levels because patient serum samples had been collected for a larger number of subjects. Measurement of IGF-I in plasma and serum is similar but not identical. Serum has been shown to have a higher overall concentration of metabolites than plasma, and certain analytes are easier to detect in serum^25^,^26^. With respect to IGF-I, serum has been shown to yield 5–10% higher IGF-I levels than plasma when measured^27^.

Since there are currently no reliable molecular predictors for POD, we were interested in assessing the utility of IGF-I as a potential candidate. We studied a relatively healthy and well-educated (D 14.9 ± 3.2 years, ND 14.6 ± 3.2 years) population of non-demented, elderly subjects so we could understand the relationship of this protein with delirium without the confounding effect of preexisting cognitive impairment. In fact, our careful exclusion of dementia and cognitive complaints along with its high education level suggest our population had high cognitive reserve, which is known to be an important protective factor for dementia^28^. Individuals with high cognitive reserve can accumulate the underlying pathophysiology of dementia without overt cognitive symptoms of dementia or delirium^28^. This suggests that some individuals with low IGF-I levels in our study might have been able to tolerate an underlying pathophysiological change without developing delirium. The high cognitive reserve of our sample may also make it unrepresentative of the general community population. Taken together with prior literature, our findings suggest that low IGF-I might instead be a biomarker for some other underlying neurophysiological condition that is actually the delirium risk factor, such as a neurodegenerative process.

There was considerable overlap in the distributions of serum IGF-I levels between groups. Some individuals with higher IGF-I levels developed delirium whereas many individuals with lower IGF-I levels did not. This was reflected by the failure of IGF-I concentration to achieve statistical significance as a predictor of POD. Our sample size was comparable with the largest published studies; however, the 95% confidence interval on the IGF-I adjusted odds ratio did not exclude unity. As such, our sample size was inadequate for a conclusive study. The question of statistical power and whether the sample size was adequate hinges on the clinical issue of whether the 95% confidence interval excludes a clinically relevant difference—what is the largest difference that would be considered clinically irrelevant, is it 30% or is it less than 30%? ROC analysis (not shown) found that overall total IGF-I levels, based on our assay, are a poor predictor of POD, and that sensitivity + specificity was maximized at IGF-I = 85 ng/ml corresponding to sensitivity =0.90, specificity =0.25, NPV =0.90 and PPV =0.30 for POD (using the observed 27% prevalence in this study).

Furthermore, our data do not support any association between total IGF-I levels and severity of delirium as measured by the DRS-R98. And the DRS-R98 is a more sophisticated measure of delirium phenomenology than the simpler diagnostic approaches of incidence that prior reports have used.

It is also possible that total IGF-I concentration is only weakly associated with POD, that confounders mask the effect of total IGF-I, or that delirium associated with acute medical illness is more linked to an IGF-I relationship than is the brief biological stressor of elective orthopedic surgery. A weak association as well as generally inadequate sample sizes could explain why previous studies have not consistently found a statistically significant association between IGF-I levels and risk of POD. Compared with five studies that have looked at delirium in acutely ill medical patients, only two studies, Lemstra *et al.* and Cerejeira *et al.*, have investigated IGF-I levels in POD and neither has found an association^17^,^20^. Of these two, only Cerejeira *et al.* excluded patients with baseline dementia or cognitive dysfunction.

The role of IGF-I binding proteins (IGFBP) in POD is both important and understudied. IGF-I exists in circulation bound to one of six different IGFBPs^29^,^30^. The assay in our study measured total IGF-I (bound + unbound) rather than a specific fraction. No previous report has examined the relationship of a specific fraction of IGF-I to POD^16^,^17^,^18^,^19^,^20^,^21^,^22^. Measuring total IGF-I levels offers only a limited picture, as differences in IGFBP profile can produce changes in the biologically active, unbound free-fraction of IGF-I^31^. Diseases like renal failure and diabetes can cause an increase in circulating IGFBPs, while certain conditions that lower IGFBP-I and -II can increase the free IGF-I levels^32^,^33^. Additionally, serum IGFBPs degrade easily, and excessive freeze/thaw cycles or suboptimal storage conditions can introduce error into an analysis^34^. Studies tailored to measure free IGF-I or IGFBPs may provide a clearer picture of IGF-I’s true influence.

In addition, numerous variables including circadian rhythm, nutritional state, renal function and hormone and insulin levels have been shown to affect IGF-I expression^35^,^36^,^37^,^38^,^39^,^40^. These variables were not addressed in our study, and therefore may confound our results.

An ideal predictor should be both a sensitive and specific detector of a disease state^41^. Unfortunately, preoperative total IGF-I levels in our study fail to reliably rule-in or rule-out the development of POD at IGF-I cutoffs outside of either the high or low extremes. Further, assay standardization and validity for determining abnormal cutoff values would need to be developed across a large and diverse community sample population. Alternatively, a meta-analysis could be used to answer the question IGF-I’s prognostic value in POD. However, at present there are not enough suitable candidate studies to pool, as only our study and Cerejeira *et al.* focus on healthy patients in an elective surgical setting. If in the future other research groups use compatible study designs to publish on this research question, then a meta-analysis could become a viable tool.

In conclusion, our study failed to show a statistically significant difference in mean IGF-I total serum levels between post-operative delirious and non-delirious subjects, and evidence for IGF-I as a predictor for POD was not found. There are currently no studies supporting a clear association between total IGF-I serum levels and POD. We recommend that future work evaluating a relationship between circulating IGF-I and POD investigate free IGF-I while controlling for potential confounders.

## Materials and Methods

### Subjects and Clinical Procedures

This prospective study enrolled 106 elderly subjects undergoing elective knee replacement surgery. Subjects were evaluated for delirium prior to surgery and on postoperative days 2 and 3. Subjects were recruited from Duke University Medical Center and the Durham Veterans Affairs Medical Center. Inclusion criteria included age ≥65 years, prior scheduling for elective knee replacement, and written informed consent per the Duke University Health System IRB (DUHS IRB). Subjects were excluded from the study if they had 1) ongoing major depression 2) psychosis or active alcohol or substance abuse within the last three months 3) preexisting delirium, dementia or cognitive impairment defined as an MMSE score less than 24 or 4) any clinically significant neurologic disorder. The experimental protocol was approved by DUHS IRB and was carried out in accordance with its guidelines.

Preoperative cognitive function was assessed using the Mini-Mental State Examination (MMSE)^42^, a chart review for prior diagnoses of dementia, and a semi-structured interview with the subject and family member. Subjects were assessed by trained research team members for preoperative delirium using the four-item Confusion Assessment Method (CAM)^43^ and the 16-item Delirium Rating Scale-Revised-98 (DRS-R98)^44^. The DRS-R98 is a well-validated tool that can be used to both diagnose delirium (using the 46-point Total scale) and measure the severity of delirium (using the 39-point Severity scale)^45^.

Subjects were then evaluated for delirium and delirium severity again following surgery on postoperative days 2 and 3 using the CAM and DRS-R98, respectively. Delirium was diagnosed by the study psychiatrist in accordance with the criteria described in the Diagnostic and Statistical Manual for Psychological Disorders, 4^th^ edition (DSM-IV) based on an in-person assessment and a review of the daily CAM, DRS-R98 and medical records. Additionally, subject American Society of Anesthesiologists Physical Status Classification (ASA) scores were obtained from anesthesiology charts. ASA scores estimate preoperative physical health on a six- (previously four-) point scale based on the presence and severity of systemic disease^46^,^47^.

### Laboratory Methods

Of the original 106 subjects enrolled in this study, serum was available from 98 individuals. After each assessment (baseline, post-op day 1 and post-op day 2) 15–20 mL of blood was collected from each patient (Three EDTA-containing tubes for plasma and peripheral blood mononuclear cell isolation and one plain tube for serum isolation). These products were flash frozen in liquid nitrogen and stored in a −80 °C freezer until use.

Preoperative IGF-I total serum levels were measured using Human IGF-I Quantikine ELISA kits (R&D Systems, Minneapolis, USA) and values expressed as concentrations (ng/ml). The ELISA was conducted according to the manufacturer’s instructions, and the subject samples were assayed in duplicate and values averaged. All duplicates possessed <10% coefficient of variation.

### Statistical Analysis

Data were analyzed using the SAS version 9.3 (SAS Inc., Cary, NC USA) statistical package. Categorical variables were summarized as frequencies and percentages, and Fisher’s exact test was used to compare study groups on categorical variables. Normally distributed baseline variables were summarized as mean ± SD and D and ND groups compared using a 2-sided, 2-sample t-test taking into account equal or unequal variances. The distribution of baseline IGF-I concentrations among non-delirious subjects was right-skewed with extreme values rendering the normality assumption untenable. However, taking logarithms sufficiently normalized the non-delirious distribution and retained approximate normality in the delirious group to enable a comparison using a 2-sample t-test. Consistent with the logarithmic analysis, geometric means (GM) and standard deviations (GSD) were reported as sample statistics—note that GM × GSD approximates the arithmetic mean. A 95% confidence interval on the GM ratio of baseline IGF-I concentrations was obtained from the logarithmic analysis. In addition, we obtained the non-parametric Hodges-Lehmann median difference estimate and a 95% distribution free (Moses) confidence interval.

POD was defined as presence of delirium on postoperative day 2 and/or 3. POD was analyzed as a binary outcome (present/absent) and as a measurable variable based on the DRS-R98 POD severity scale. Univariate logistic regression analysis was used to evaluate pre-operative subject characteristics as predictors of POD (presence/absence). Variables in the univariate analysis with p ≤ 0.15 were entered into a multivariate analysis. In the subset of subjects with POD, association of DRS-R98 Total score and Severity score with baseline variables was assessed using univariate least squares regression for continuous baseline variables and a 2-sample t-test for categorical variables. Baseline variables significant at p ≤ 0.15 were entered into a multivariate analysis (not shown).

## Additional Information

**How to cite this article**: Yen, T. E. *et al.* Association between Serum IGF-I levels and Postoperative Delirium in Elderly Subjects Undergoing Elective Knee Arthroplasty. *Sci. Rep.* **6**, 20736; doi: 10.1038/srep20736 (2016).

## Figures and Tables

**Table 1:** Baseline (preoperative) demographic variables, subject characteristics and IGF-I levels.

Variable	Non-Delirious (n = 76)	Delirious (n = 22)	P-value^1^
Demographic and Clinical Characteristics			
Age (years)	73.7 ± 5.2	72.5 ± 4.4	0.351
Sex (female)	50%	59%	0.479
Caucasian	92.1%	81.8%	0.225
BMI	31.1 ± 7.3	31.7 ± 7.6	0.692^a^
Education (years)	14.9 ± 3.2	14.6 ± 3.2	0.663
ASA score of 3^†^	60.5%	54.6%	0.631
Mini-Mental State Exam, median (range)	30 (24, 30)	29 (26, 30)	0.161^b^
Hemoglobin (g/dl)	13.4 ± 1.6	13.3 ± 1.1	0.718
Preoperative % oxygen saturation	96.9 ± 1.6%	97 ± 1.7%	0.800
Number of preoperative comorbidities	3.4 ± 1.8	3.2 ± 1.4	0.711
Frequency of comorbidities:			
Coronary artery disease	15.8%	27.3%	0.226
Diabetes	10.5%	22.7%	0.159
Hypertension	75.0%	81.8%	0.582
Hyperlipidemia	44.7%	45.5%	1.000
Obstructive sleep apnea	9.2%	31.8%	**0.014**
Benign prostatic hypertrophy	19.7%	9.1%	0.345
Incontinence	11.8%	9.1%	1.000
Gastroesophageal reflux disorder	32.9%	31.8%	1.000
Preoperative IGF-I serum level (ng/mL)			
Mean ± SD	68.4 ± 31.6	57.0 ± 18.6	**–**a
Geometric mean (Geom. SD)	62.3 (1.10)	53.6 (1.07)	0.146^b^
Median (range)	65.9 (17.2, 204)	62.6 (19.6, 87.3)	0.141^c^

^†^All subjects had a preoperative ASA score of either 2 (mild systemic disease) or 3 (severe systemic disease).

^1^Two-sample t-test (equal or unequal variances as appropriate) on continuous variables for which normality assumption is tenable, unless indicated otherwise; Fisher’s exact on categorical variables.

^a^Two-sample t-test p = 0.037, but outliers make normality assumption untenable.

^b^Two-sample t-test on logarithms.

^c^Two-sample (rank-sum) Wilcoxon test.

**Table 2:** Results of Univariate and Multivariate Analysis.

Variable	Univariate^1^		Multivariate	
	Odds ratio with Confidence Interval	P-value	Odds ratio with Confidence Interval	P-value
Obstructive sleep apnea	4.60 (1.40, 15.1)	**0.012**	4.06 (1.21, 13.7)	**0.024**
Diabetes	2.50 (0.73, 8.62)	0.147	2.08 (0.56, 7.66)	0.273
IGF-I Level	0.983 (0.962, 1.004)	0.108	0.985 (0.964, 1.007)	0.183

Results of the univariate logistic regression analyses investigating 19 variables as potential predictors of post-operative delirium incidence and of the multivariate analysis using the 3 variables significant at p ≤ 0.15 in the univariate analysis. Significant results are bolded.

^1^Age, sex, race, ASA score, MMSE score, education, BMI, preoperative hemoglobin and oxygen saturation, number of preoperative comorbidities, CAD, hypertension, hyperlipidemia, BPH, incontinence and GERD had p-values > 0.15 in univariate analysis and were not included in the multivariate analysis. Only OSA, Diabetes and IGF-I levels were included in the multivariate analysis.
